# Efficiency and herding analysis in gold-backed cryptocurrencies

**DOI:** 10.1016/j.heliyon.2022.e11982

**Published:** 2022-12-01

**Authors:** Emna Mnif, Bassem Salhi, Lotfi Trabelsi, Anis Jarboui

**Affiliations:** a Department of Finance, University of Sfax, Sfax, Tunisia; b Department of Accounting, College of Business Administration, Majmaah University, Majmaah, 11952, Saudi Arabia; c Department of Finance and Accounting, University of Sfax, Sfax, Tunisia; d Department of Management, University of Sfax, Sfax, Tunisia

**Keywords:** Gold-backed cryptocurrencies, Sharia-compliant, Efficiency index, Herding bias, Generalized Hurst exponent, COVID-19

## Abstract

This study analyses and compares the behavior of the gold-backed, conventional cryptocurrency, and gold markets capable of detecting the existence of herding and deducing the efficiency degree. In addition, this empirical work tried to examine the COVID-19 pandemic's influence on both cryptocurrency performances. This work developed a new method that discloses herding biases using persistence and efficiency metrics. Besides, this paper investigated the nonlinear dynamic properties of the gold-backed, conventional cryptocurrencies and Gold by estimating the Multifractal Detrended Fluctuation Analysis (MFDFA). It also assessed the inefficiency of these markets through an efficiency index (IEI) and tested the effect of COVID-19 on their dynamics. The findings of this investigation indicate that the gold-backed cryptocurrency (X8X) is the most efficient market in the long-term trading market. However, the conventional cryptocurrency market (Bitcoin) is the most efficient on the short trade horizon. Besides, gold-backed cryptocurrency markets present a smaller level of herding behavior than conventional cryptocurrencies on tall scales. Nevertheless, we noted the positive and negative effects of the pandemic on each cryptocurrency market dynamics. To the best of the authors' knowledge, this study is the first investigation that uses multifractal analysis to quantify the impact of the COVID-19 spread on gold-backed cryptocurrencies and detects the presence of herding behavior.

## 1 Introduction

Since 2009, Bitcoin has been the main focus of investment and academic research. This interest has been widened to cover cryptocurrencies and blockchain in recent years.

Conventional cryptocurrencies are virtual currencies often based on decentralized networks built on blockchain and not backed by any physical asset. However, "gold-backed cryptocurrencies" are novel technologies indexed to the value of a real object (Gold). These digital coins have inherent worth in addition to being on a distributed ledger and hence are simple to trade, which makes them exceptional to conventional cryptocurrencies (Aloui et al., 2021).

Besides, these technologies are similar to stablecoins as they are invented to reduce market risks and deal with excessive volatility. »

From a behavioral standpoint, cryptocurrencies generate a vast amount of data that depicts the preferences of investors (Böhme et al., 2015). Because of the bearishness of crypto assets in 2018, most investors are perceived to be buy-hold operators (Vidal-Tomás et al., 2019). Due to the lack of financial fundamentals, cryptocurrency is vulnerable to sentiment issues and behavioral biases (Kaiser and Stöckl, 2019). Some events like disasters or pandemic diseases like COVID-19 may incite this behavior. Nowadays, COVID-19 provokes more and more anxiety and panic among investors and traders. In particular Muslim investors who attempt to satisfy their religious needs. Muslim populations are increasing to count more than 1.9 billion. Their capital and liquidity are more accumulated, drawing the attention of researchers to satisfy their demand for cryptocurrencies.

From an Islamic view, most cryptocurrencies are not backed by a tangible asset and lack fundamental values that deny their compatibility with sharia laws (Siswantoro et al., 2020). Therefore, new innovative technological applications exploited the existing blockchain to satisfy the religious requirements of some investors by backing this technology to Gold and other currencies such as X8X and HelloGold. Unlike conventional cryptocurrencies, Sharia-compatible ones are based on quantitative financial elements that support their prices. Gold-backed cryptocurrencies use physical Gold as the underlying asset. They are created to be a less volatile investment as their prices are linked to Gold. For these reasons, we propose to compare the inefficiency and herding bias level in the conventional, gold-baked cryptocurrencies and gold markets.

To our knowledge, the efficiency of gold-backed cryptocurrencies and their response during the crisis have not been investigated despite their importance. Motivated by this gap in the literature, this paper aimed to explore the presence of herding bias and the efficiency level through a multifractal detrended fluctuation analysis during the novel coronavirus crisis. The inefficiency of cryptocurrencies has been chiefly considered from a behavioral perspective during the crisis. Several studies have examined the efficiency and the presence of herding relationships capable of changing future price dynamics.

Banerjee (1992) defines herding behavior as the act of replicating what others do rather than using one's personal information. This behavior bias leads to excessive uncertainty (Humayun Kabir and Shakur, 2018). As a result, repeated behavioral patterns produce speculative bubbles and crashes. In finance, herding bias can be created by unintended behavior provoked by an event that simultaneously pushes traders and investors to sell and buy the same asset (Lakonishok et al., 1992). Similarly, this bias can relate to purposeful variables such as reputational concerns (Scharfstein and Stein, 1990) or informational cascades (Avery and Zemsky, 1998). This bias was investigated in cryptocurrency markets (Ballis and Drakos, 2019; Mnif, Jarboui, and Mouakhar, 2020a; Aloui et al., 2021), commodity markets (Demirer et al., 2015), and stock markets (Chiang and Zheng, 2010).

In Sharia-compliant markets, Chaffai and Medhioub (2018a) partially proved the existence of herding behavior in some stock markets. At the same time, Mnif et al. (2019) justified its presence in the Dow Jones Islamic markets. Gharib et al. (2021) have explored the contagion effect on gold markets and their response to the COVID-19 pandemic. Particularly, Yousaf and Yarovaya (2022) studied the contribution of Islamic gold-backed cryptocurrencies in reducing portfolio risks during the COVID-19 pandemic. While Jalan et al. (2021) have explored gold-backed cryptocurrencies and compared their performance with Bitcoin and Tether during the pandemic. They showed that these gold-backed cryptocurrencies were not considered safe-haven assets during this crisis.

Most researchers considered that the main problem with cryptocurrencies is related to their exceptionally high volatility resulting from herding intensity, especially during the COVID-19 pandemic (Evrim Mandaci and Cagli, 2021). Therefore, by basing their value on Gold, investors will be reassured and place their money with more security. For these reasons, we propose to study the behavior of gold-backed cryptocurrencies, conventional cryptocurrencies, and Gold by comparing their efficiency and the herding intensity during the COVID-19 pandemic.

Consequently, the proposed methodology is based on the following assumptions:

H1 Gold-backed cryptocurrencies are less inefficient than Gold and conventional cryptocurrency markets before the COVID-19 pandemic;

H2 Gold-backed cryptocurrencies are more efficient than the other studied markets after the COVID-19 pandemic;

H3 The COVID-19 pandemic arouses a herding level among cryptocurrency market investors.

This paper contributes to the current literature in a range of the following aspects. First, it investigated the dynamics of a new family of cryptocurrencies with distinct characteristics. It explored the efficiency of cryptocurrencies with quantifiable financial fundamentals backed by Gold and currencies. Second, this paper used a novel pandemic virus event and two international cryptocurrencies. The first category is distinguished by its backing to Gold, while the other is characterized by its considerable trading volume and market capitalization. Third, this study relied on a sophisticated method to detect the presence of herding behavior and assess the efficiency level in these markets during this pandemic. Fourth, It explored the multifractality that existed during and before the COVID-19 outbreak. As a result, our study covers the entire period and provides a thorough picture of these two types of cryptocurrency fluctuations.

This research would be helpful for traders, investors, and policymakers.

This work is structured as follows: A summarized literature review was gathered and reviewed in the second section. The third section described the data, while the fourth and fifth sections provided the methodology and the results, respectively. The last part draws the main conclusions of the study.

## 2 State of knowledge and hypotheses development

Financial stability and market efficiency were the focus of academic research and marketers' analyses. According to the efficient market theory, all available information is used in setting values and reflected immediately in investors' behavior (Fama, 1970). Therefore, it is essential to note that a decrease in market inefficiency implies that its prices are less forecastable. In contrast, increased market inefficiency indicates that the market is more predictable with available information. Over the last few years, many kinds of research have been conducted to estimate the efficiency level of the cryptocurrency market. Some studies attempted to test the validity of the efficiency of the cryptocurrency markets (Bariviera, 2017; Jiang et al., 2018; Vidal-Tomás and Ibañez, 2018; Nan and Kaizoji, 2019). Their results are similar and reject the efficiency hypothesis. Another strand of research has studied the evolving inefficiency of cryptocurrency markets (Bariviera, 2017; Mnif and Jarboui, 2021). For this purpose, they both use technical and fundamental tools. Financial innovation tools such as mathematics-based models can so spell out stability conditions. Previous theories based on the Gaussian distribution have proven ineffective in forecasting the dynamics of capital markets (Fry and Cheah, 2016).

Nevertheless, multifractal models are more robust in detecting financial market stability (Oświcimka et al., 2006). The fractal theory was first introduced by B. B. Mandelbrot (1975), who defined fractals as complex geometrical bodies with one scaling feature. This theory was employed in the Sharia-compliant market analysis by Bouoiyour et al. (2018) and Mensi et al. (2017) using the MFDFA to compare the Islamic market efficiencies. For these reasons, this work adopts the fractal theory to determine the cryptocurrency market efficiency by the scale and define an inefficiency index to compare their various behaviors.

SARS epidemic disease (Kostoff, 2011) and Malaria (Cervellati et al., 2018) have been explored into the impact of infectious illnesses and pandemics. Furthermore, Bennett, Chiang and Malani (2015) and Claessens, Dell'Ariccia, Igan and Laeven (2010) discussed their impact on financial and economic dimensions.

The recent COVID-19 pandemic has provoked intensive disturbance on financial stability and the global economy. In particular, several studies developed the COVID-19 effect on the efficiency of stock markets, such as Al-Awadhi et al. (2020) and Ashraf (2020), who demonstrated that the capital markets responded negatively to the growth of confirmed cases and fatalities. In the same vein, the stability of financial markets was explored by several works (Baker et al., 2020; Ozili and Arun, 2020; Baek et al., 2020), who confirmed the negative impact of this pandemic on the capital market stability. Tanin et al. (2021) have explored Gold's efficiency and ability to be considered a safe haven during the pandemic in gold markets. In the same trend, several works have confirmed the effect of the pandemic in increasing cryptocurrency market inefficiency. This influence was investigated by Salim Lahmiri and Bekiros (2020), who compared informational efficiency and stability. In gold-backed cryptocurrencies, Jalan et al. (2021) have examined the performance of these assets during the pandemic. However, the evolving inefficiency level of gold-backed cryptocurrencies has not been widely explored during the pandemic. We propose to fill this gap in this paper by testing the following hypotheses:

H1 Gold-backed cryptocurrencies are less inefficient than Gold and conventional cryptocurrency markets before the COVID-19 pandemic;

H2 Gold-backed cryptocurrencies are more efficient than the other studied markets after the COVID-19 pandemic.

Besides, most studies agreed that COVID-19 and the 2008 crisis increased the herding behavior among investors (Mnif et al., 2020b; Ferreruela and Mallor, 2021; Shrotryia and Kalra, 2021). In addition, the presence of herding behavior in periods of pandemics in European stock markets was investigated by Espinosa-Méndez and Arias (2020). Their results showed that this crisis increased the herding bias in the capital market of France, Italy, Germany, the United Kingdom, and Spain. This bias was also explored in oil and energy stock markets during two crisis periods; the COVID-19 pandemic and the global financial crisis. Their findings showed that herding behavior increased in both periods of crisis in these markets, especially in extreme cryptocurrency market losses. However, Yarovaya et al. (2020) demonstrated that this event does not amplify herding in cryptocurrency markets during this pandemic.

The established relationships between multifractality and herding were further developed in other empirical research, such as those offered by Cajueiro and Tabak (2009) and Fernández-Martínez et al. (2017). They underlined a relationship between market persistence and the presence of herding behavior. This link was also explored by Mnif, Jarboui and Mouakhar (2020a) and Wasiuzzaman and Haji Abdul Rahman (2021) to determine the efficiency and the existence of this bias in the cryptocurrency market. They argued that the presence of this bias leads investors to behave in the same way. In other words, investors will buy simultaneously or sell simultaneously, leading to persistence in the price dynamics (H > 0.5) and therefore creating long memory in the prices' shapes. Following these researchers, this work proposes identifying herding behavior by using the efficiency level through the inefficiency index (IEI).

Although this extensive literature on the repercussion of the pandemic on the financial markets and investor behavior, this impact was not well studied when it comes to gold-backed cryptocurrencies. For these reasons, this paper proposes to fill this gap by testing the following hypothesis:

H3 The COVID-19 pandemic arouses a herding level among cryptocurrency market investors.

Therefore, this study's major contribution was exploring the role of the current coronavirus pandemic in increasing the gold-backed cryptocurrency market inefficiency and herd behavior. The implications of this research are fundamental for cryptocurrency traders and policymakers in understanding and anticipating financial market outcomes during the coronavirus crisis.

## 3 Data

The data used in this work include clinical and financial data. The financial data include returns of Gold and four cryptocurrencies, namely Bitcoin (BTC), Ethereum (ETH), X8X (X8X), and HelloGold (HGT), retrieved in daily frequencies before the onset of the COVID-19 (Table 1) and after this pandemic outbreak (Table 2) based on their availability from the coinmarketcap site (www.coinmarketcap.com). Gold series is extracted from the Yahoo finance site web (www.finance.yahoo.com). The period of the gold data is selected in parallel with the most extended available data of gold-backed cryptocurrencies.

**Table 1:** Summary statistics of the studied cryptocurrency returns.

	Time period	Min	Max	Mean		SD	Skewness	Kurtosis	P (J-B)	Obs
Panel 1: Before the COVID-19 pandemic										
HelloGold	12/10/2017–30/12/2019	-0.9681	0.9856	-0.0031		0.1121	0.1169	17.1768	<2.2e-16	803
X8X	22/09/2018–30/12/2019	-0.7838	0.5183	-0.0003		0.0637	-2.3067	52.9062	<2.2e-16	511
Bitcoin	28/04/2013–30/12/2019	-0.0993	0.0985	0.0005		0.0166	-0.2671	5.2787	<2.2e-16	1931
Ethereum	07/08/2015–30/12/2019	-0.5697	0.1773	0.0009		0.0309	-3.5856	73.9632	<2.2e-16	1607
Gold	12/10/2017–30/12/2019	-0.0101	0.0098	-0.0001		0.0023	0.0228	2.5252	<2.2e-16	803
Panel 2: After the COVID-19 pandemic										
HelloGold	31/12/2019–12/03/2021	-0.2437	0.1901	0.0007	0.0456		-0.1367	5.1254	<2.2e-16	423
X8X	31/12/2019–12/03/2021	-0.2398	0.1533	0.0003	0.0453		-0.7604	4.6941	<2.2e-16	423
Bitcoin	31/12/2019–12/03/2021	-0.2159	0.0776	0.0018	0.0193		-0.7604	38.3193	<2.2e-16	423
Ethereum	31/12/2019–12/03/2021	-0.2574	0.1012	0.0025	0.0255		-2.3181	25.9345	<2.2e-16	423
Gold	31/12/2019–12/03/2021	-2.56e-02	1.7e-02	-8.03e-05	0.0047		-0.7816	3.7959	<2.2e-16	423

Clinical data comprise the growth of the daily confirmed cases and the daily new deaths due to COVID-19 worldwide from Worldmeter, covering the period extending from 31 December 2019 to 12 March 2021.

**Table 2:** Generalized Hurst exponent (GHE) estimates for -5<q < 5.

q	Bitcoin		Ethereum		HelloGold		X8X		Gold	
	S < 30	S > 30	S < 30	S > 30	S < 30	S > 30	S < 30	S > 30	S < 30	S > 30
-5	0.9578	0.7175	0.8336	0.8104	0.7146	0.6154	0.7193	0.5964	1.0751	0.6225
-4	0.9053	0.6967	0.7964	0.7934	0.6784	0.5891	0.6813	0.5746	0.9966	0.5997
-3	0.8399	0.6744	0.7571	0.7733	0.6312	0.5592	0.6364	0.5486	0.8850	0.5720
-2	0.7653	0.6560	0.7165	0.7497	0.5759	0.5276	0.5894	0.5191	0.7464	0.5386
-1	0.6878	0.6474	0.6735	0.7225	0.5158	0.4964	0.5489	0.4920	0.6185	0.4986
0	0.6147	0.6400	0.6242	0.6925	0.4538	0.4643	0.5107	0.4801	0.5275	0.4504
1	0.5632	0.6160	0.5517	0.6618	0.3888	0.4234	0.4327	0.4746	0.4638	0.3911
**2**	**0.5406**	**0.5749**	**0.4089**	**0.6329**	0.3068	**0.3623**	**0.3066**	**0.4520**	**0.4178**	**0.3193**
3	0.5279	0.5281	0.1881	0.6071	0.2161	0.2888	0.2004	0.4169	0.3870	0.2440
4	0.5043	0.4861	-0.0069	0.5848	0.1486	0.2243	0.1351	0.3813	0.3674	0.1790
5	0.4727	0.4525	-0.1292	0.5658	0.1050	0.1763	0.0950	0.3503	0.3544	0.1295

**Figure 1:**
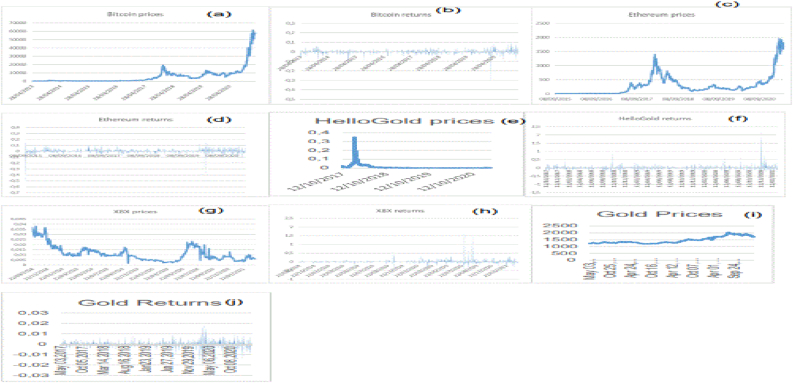
Bitcoin (a, b), Ethereum (c, d), HelloGold (e, f), X8X (g, h), and Gold (i, j) prices and returns.

The cryptocurrency returns are calculated in equation 1 as follows

(Equation 1) $$r_{t}=\mathrm{Log}\left(\frac{p_{t}}{p_{t-1}}\right)$$

rt and Pt are, respectively, the cryptocurrency returns and prices at date t and presented in Figure 1. Accordingly, the cryptocurrencies' prices show high volatility. Therefore, the effect of COVID-19 is observed in all the cryptocurrency markets.

Table 1 reports the asymmetric characteristics of cryptocurrency price distribution reflecting the inefficiency level in these markets. The skewness and Kurtosis tests indicate that all series distributions are not Gaussian.

## 4 Methodology

The proposed methodology consists of two main parts. In the first one, we compared the dynamics of the cryptocurrencies and highlighted the trading opportunities in the short and long term. In the second part, we investigated the effect of COVID-19 on cryptocurrency efficiency. Firstly we compared the efficiency and the presence of herding behavior before and after the COVID-19 outbreak. Secondly, we explored the effect of the pandemic proxies on the studied cryptocurrencies.

FERNÁNDEZ-MARTÍNEZ et al. (2017) postulate that the appearance of herding behavior supposes that investors' purchasing will increase, leading to a price climb and cryptocurrency market persistence surge.

In this study, we quantify the magnitude of this behavior by referring to the level of inefficiency and using self-similarity exponents. In other words, the highest the cryptocurrency inefficiency level is, the more herding behavior exists. For this reason, we compare the evolution of these two proxies before and after the pandemic outbreak.

### 4.1 Cryptocurrencies' efficiency through multifractal analysis

The first part of the methodological study focused on examining the gold-backed, Gold, and conventional cryptocurrency dynamics by underlining the short-horizon and long-term trading opportunities. For that purpose, our research used fractal theory to determine herding behavior and then analyzed the inefficiencies of cryptocurrency markets. Previous studies link the Lévy stable condition or α, which varies from 0 to 2, to persistence patterns. In the case of α = 2, the series has a Gaussian distribution (Blumenfeld and Mandelbrot, 1997).

The MFDFA method consists of 5 steps, as explained by (Mnif, Jarboui, and Mouakhar, 2020a). The series is monofractal when H(q) is constant for all q. Otherwise, the series becomes multifractal.

This paper estimated the multifractal spectrum with various m values (m = 1, m = 2, and m = 3). Accordingly, the order is set at m = 1 to avoid overfitting problems.

The roughness of financial markets is quantified by B Mandelbrot (1963) by estimating the Holder exponent and defining the fractal dimension (d) as indicated in equation 2 and equation 3:

(Equation 2) d=2−Hwhen0<H<1

(Equation 3) andd=1.5−αwhen−0.5<α<0.5

The scaling function of the multifractal process τ(q) is concave for the multifractal and linear for the monofractal process. τ(q) can be formulated in Eq. (4) from the generalized Hurst exponent as:

(Equation 4) $$H\left(q\right)=\frac{1+\tau \left(q\right)}{q}$$

After that, we calculated the range ΔH ≡ maxq H(q) - minq H(q) and the width of the spectrum Δα ≡ maxq α(q) - minq α(q) to measure the level of multifractality which increases when either of these measures increases. Table 2 summarizes these estimated parameters for the studied cryptocurrencies.

We also fixed the scaling range at s_min_ = 10 and s_max_=(T/4) for MF-DFA as proposed by (Rizvi et al., 2014). T is the cryptocurrency series' length. In addition, we used τ_min_ = 1, and we varied τ_max_ between 5 and 20, as suggested by Kukacka and Kristoufek (2020).

This section also defined a measure of inefficiency using the generalized Hurst exponent. If IEI = 0, the market is completely efficient with no herding behavior and no persistence. The inefficiency index (IEI), as employed by Mnif, Jarboui, and Mouakhar (2020b), is denoted in Eq. (5) as:

(Equation 5) $$IEI=\frac{1}{2}\left(\left|h\left(-5\right)-0.5\right|+\left|h\left(5\right)-0.5\right|\right)$$

### 4.2 The impact of the COVID-19 pandemic on the studied markets’ behavior

In this section, we examined the effect of COVID-19 on gold-backed, conventional cryptocurrencies and gold markets. Firstly, we compared their efficiency and multifractality before and after the COVID-19 outbreak date (31/12/2020). Secondly, we estimated the effect of this pandemic on the cryptocurrency and gold returns through a system GMM approach.

As the event peak did not happen at the start date and lasted several days, this paper did not follow a classical event study methodology. It employed the system GMM approach based on the two-step estimation to provide more effective results and deal with correlation, endogeneity, and heteroscedasticity issues (Efe et al., 2016).

We regressed the cryptocurrency returns R_i,t_ on the lagged previous daily cryptocurrency predictors (R_i,t-1_), the daily growth in newly confirmed cases GC (equation 6), and the daily change in fatalities GD (equation 7) (Ftiti et al., 2021) during the period between 23 January 2020 and 12 March 2021.

(Equation 6) $$GC=\frac{{Cumulative\;cases}_{t}-{Cumulative\;cases}_{t-1}}{{Cumulative\;cases}_{t-1}}$$

(Equation 7) $$GD=\frac{{Cumulative\;deaths}_{t}-{Cumulative\;deaths}_{t-1}}{{Cumulative\;deaths}_{t-1}}$$

Therefore, we estimated the cryptocurrency returns in Eq. (8) as:

(Equation 8) $$R_{i,t\;}=\alpha_{0\;}+\alpha_{1}\;R_{i,t-1}+\alpha_{2}\;{GC}_{i,t-1}+\alpha_{3}\;{GD}_{i,t-1+}\varepsilon_{i,t}$$

It is crucial to guarantee two essential requirements for the consistency of System GMM estimations (Bond, 1991):

• The error term should not have any serial correlation. For this reason, we used the Arellano-Bond test to examine the first differenced equation's first and second-order autocorrelated disturbances for serial correlations, as detailed in Table 6.
• The instruments and the error term should also not be correlated. In this paper, we employed the Hansen test of over-identifying restrictions to check the validity of the instruments (Table 6).

## 5 Empirical results

**Figure 2:**
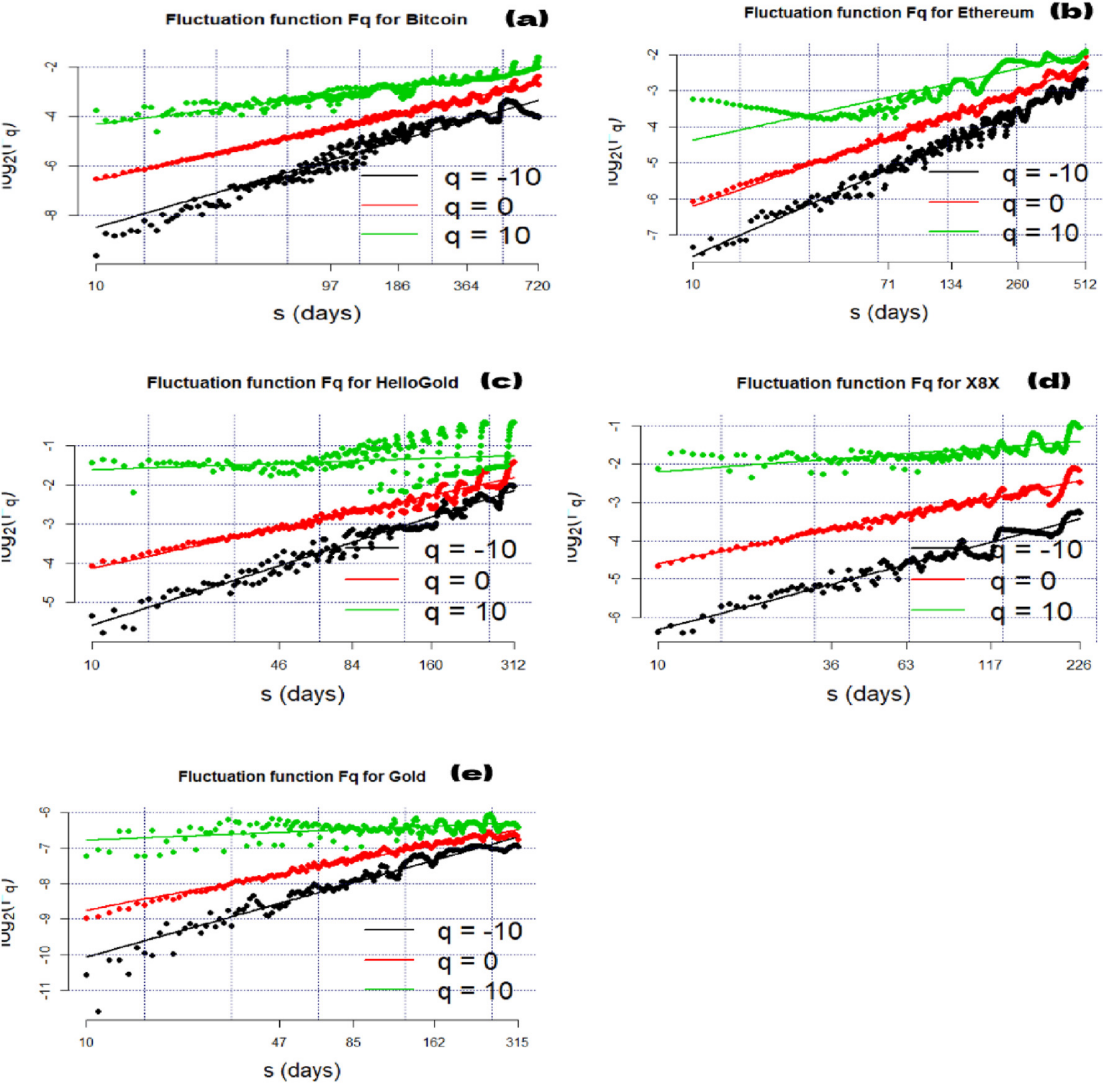
Fluctuation function for Bitcoin (a), Ethereum (b), HelloGold (c), X8X (d), and Gold (e).

After representing the prices and returns plots of the cryptocurrencies (Figure 1), this work studied the multifractal properties and efficiency dynamics of these markets. Figure 2 shows the relationship between the order of fluctuation Fq(s) and the length scale (s) in log-log plot for the Bitcoin, Ethereum, X8X, and the HelloGold cryptocurrency and Gold markets when q ranges from -10 to 10.

**Figure 3:**
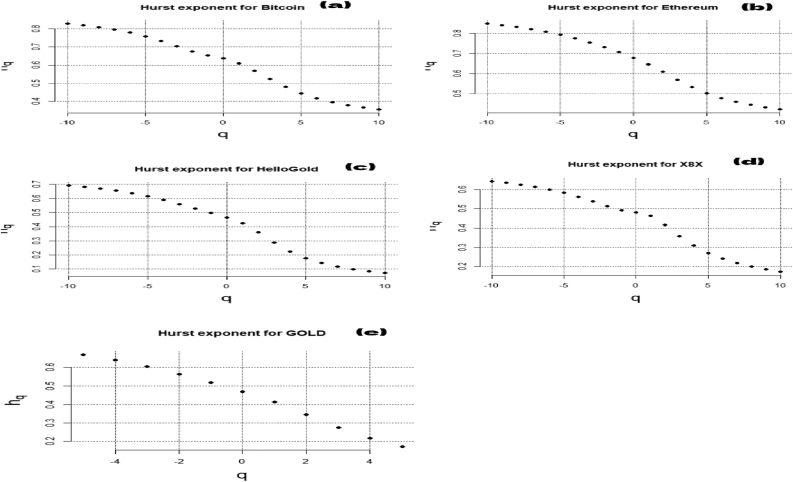
Generalized Hurst exponent for Bitcoin (a), Ethereum (b), HelloGold (c), X8X (d), and Gold (e).

**Figure 4:**
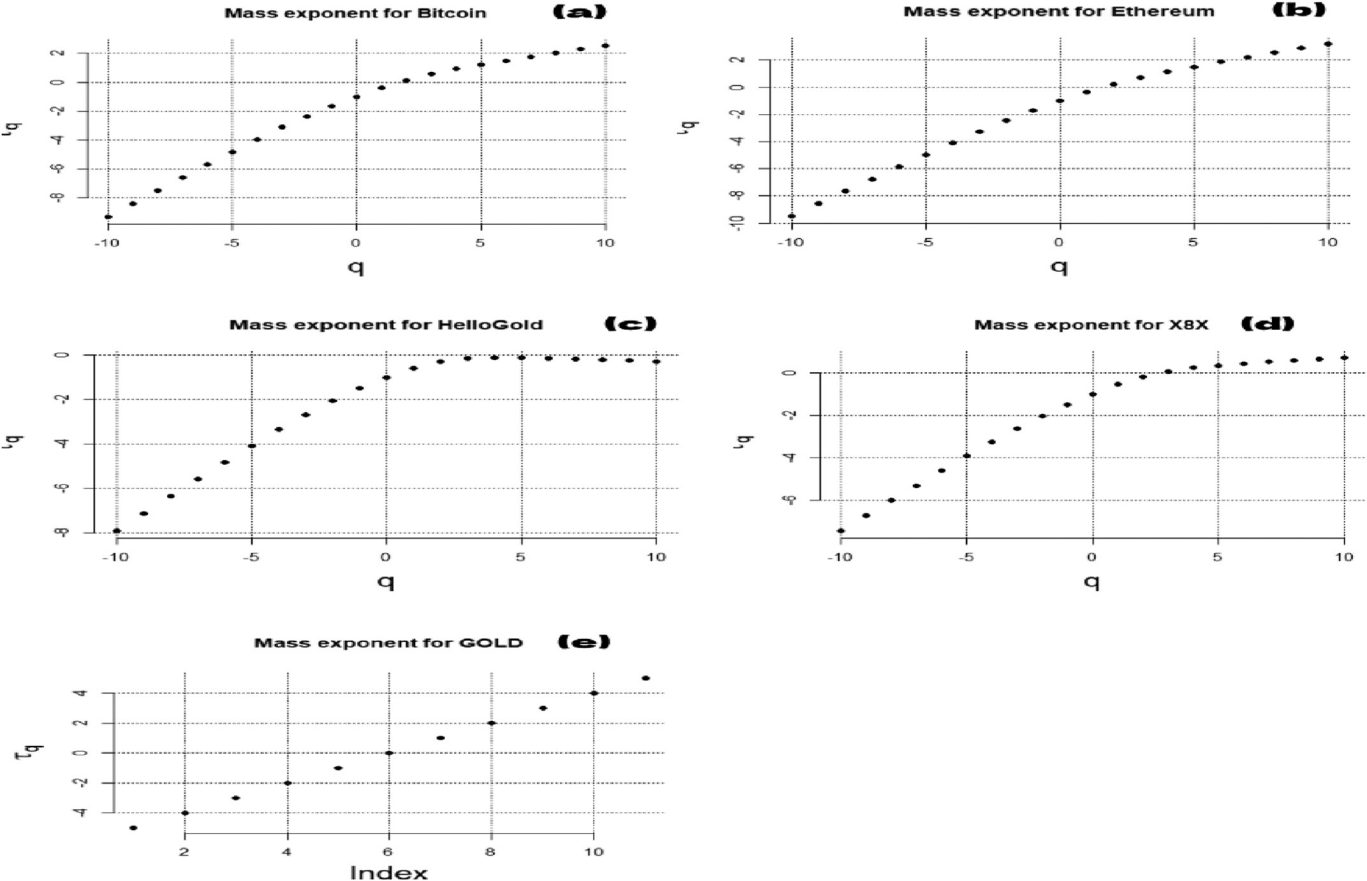
Scaling function for Bitcoin (a), Ethereum (b), HelloGold (c), X8X (d), and Gold (e).

Figure 2 reveals the slope of the regression line defining H (Hurst exponent). The q-order RMS can identify the level and the microstructure of the time series magnitude (Ghosh and Kozarevic, 2019). H(q) is not constant for all q in Figure 3, showing that all the series are multifractal. The scaling function τ(q) of the multifractal process plots in Figure 4 is concave, implying that the series used in this paper are multifractal processes.

In Figure 2, a linear behavior change is observed when the length scale is equal to 30, denoted as S∗ (approximately one month). Following Kantelhardt et al. (2002), we considered a two-time horizon corresponding to short-term trading (s < S∗) and long-term trading (s > S∗). The choice of S∗ = 30 is consistent with most studies on financial market dynamics.

**Figure 5:**
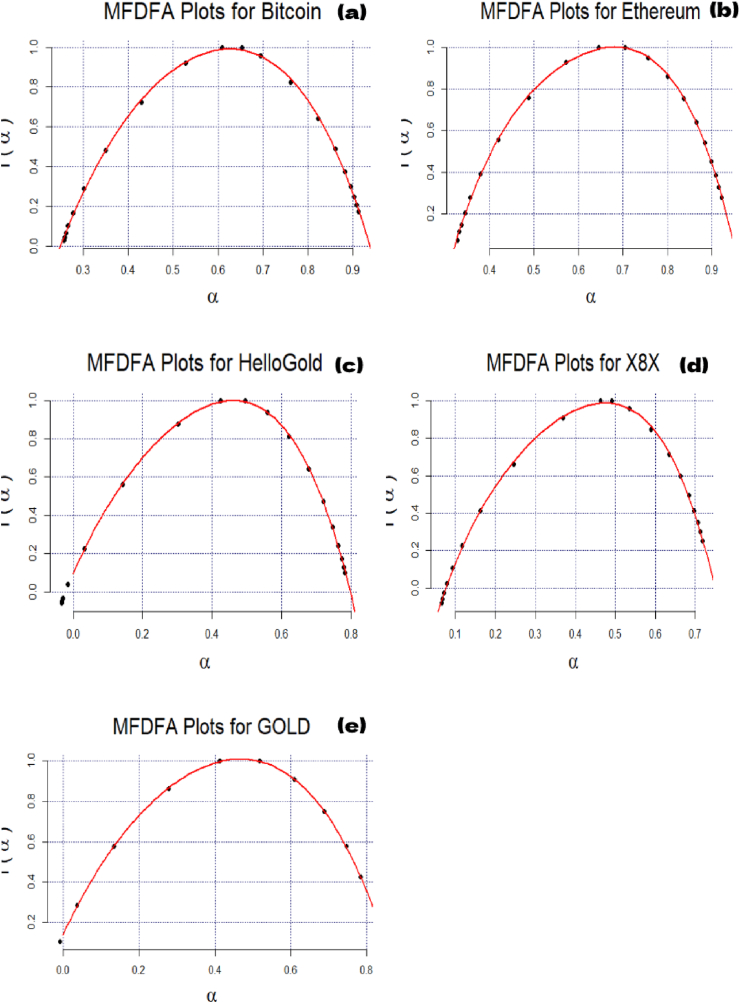
Multifractal spectrum for Bitcoin (a), Ethereum (b), HelloGold (c), X8X (d), and Gold (e).

The generalized Hurst exponent results are summarized in Table 2. The results in Figure 5 confirm our assumption of multifractality for the studied series.

We used negative q in Table 2 to explain the impact of small price variations and positive q for large variations. The multifractality of the cryptocurrency's series is confirmed in Table 3 because Hq depends on q. Table 2 shows that the persistence of the cryptocurrencies (Hq > 0.5) is observed in short-term trading when q > (−2), especially for the conventional cryptocurrencies (Bitcoin and Ethereum). Consequently, these fluctuations are caused by herding behavior in the short term (Ghosh and Kozarevic, 2019). The generalized Hurst exponent Hq () varies moderately when q changes for both scales. In q = 2, the Hurst exponent H= H (q = 2)-1 is around 0.7 in the short term, which is higher than 0.5, indicating that the Sharia-compliant cryptocurrencies are inefficient and are anti-persistent in the short horizon trading. These results align with those of Lahmiri and Bekiros (2019). However, they have become more efficient in long-term trading. In the case of conventional cryptocurrencies, the Hurst exponent H= H (q = 2)-1 is around 0.5, especially for Bitcoin, showing that these markets are likely to be more efficient than gold-backed cryptocurrencies. The literature has not investigated the efficiency of gold-backed cryptocurrencies through multifractality. However, the efficiency of the Islamic stock markets and the existence of herding behavior have been explored by several studies.

**Table 3:** Short-term trading (S < 30).

	Δα	Δ H_q_	Hurst (mean)	D	IEI	Rank
Bitcoin	0.8215	0.4851	0.6708	1.3291	0.2425	1
Ethereum	1.5677	0.9273	0.4955	1.5044	0.4636	5
HelloGold	0.9288	0.6096	0.4304	1.5695	0.3048	2
X8X	0.8059	0.6243	0.4414	1.5585	0.3121	3
Gold	1.31	0.7207	0.6217	1.3782	0.3603	4

Ali et al. (2018) indicated that the studied Islamic markets have greater efficiency than conventional markets. They attributed this result to the laws of Sharia and its good governance and disclosure. Regarding the existence of herding behavior, Mnif et al. (2019) explored the Islamic stock market to detect the presence of this behavior. They indicated that this bias might be a result of a psychological component.

These works lack a quantitative assessment of herding and efficiency during pandemics. Furthermore, previous studies have not explicitly examined the impact of diseases and disasters. This paper developed these issues by quantifying the level of multifractality with the singularity spectrum, as presented in Figure 5. The width of the fractal spectrum Δα results is reported in Tables 3 and 4. The multifractality increases when Δα is expanded (Lu et al., 2013).

**Table 4:** Long-term trading (S > 30).

	Δα	Δ H_q_	Hurst (mean)	d	IEI	Rank
Bitcoin	0.4826	0.265	0.6081	1.3918	0.1325	2
Ethereum	0.3886	0.2446	0.6903	1.3096	0.1881	3
Hellogold	0.7363	0.4391	0.4297	1.5702	0.3958	5
X8X	0.4573	0.2461	0.4805	1.5194	0.1231	1
Gold	0.534	0.493	0.4131	1.5868	0.2465	4

In an inefficient market, the dynamics of the fluctuations follow a random walk behavior, and Hq () should be equal to 0.5. For this reason, based on Hq (), we calculated the inefficiency index (IEI). In other words, the larger this index (IEI) is, the less efficient the market will be. The results in Tables 3 and 4 show that:

∗In short-term trading: the efficiency values (IEI) for the short horizon are smaller than 0.5. In addition, IEI(Bitcoin)<; IEI(HelloGold) < IEI (X8X) < IEI (Gold) < IEI (Ethereum) indicates that the Bitcoin cryptocurrency is the most efficient market in the short-run trading market.

∗In the long horizon trading: the efficiency index values (IEI) for the long scale trading are smaller than 0.4. In addition, IEI(X8X) < IEI(Bitcoin) < IEI (Ethereum) < IEI (Gold) < IEI (HelloGold) indicating that the X8X cryptocurrency is the most efficient market in the long-term trading market.

These results show that all inefficiency values (IEI) range between 0.4636 and 0.1231, indicating that all studied markets are almost efficient but vary in efficiency levels. The closest value of (IEI) to zero is the most the market is efficient.

X8X and Bitcoin rank first in long and short-term horizons over the whole study period. These cryptocurrencies are then the most tempting for both speculators and traders.

Following the Hausdorff topology (Hausdorff, 1918), the level of herding bias increases when the fractal dimension decreases and moves from Douady rabbit (d = 1.38) to dendrite Julia (d = 1.18) (Ghosh and Kozarevic, 2019).

Tables 3 and 4 show that the Hurst average and the fractal dimension are associated with the q values ranging from -5 to 2. Therefore, the reported results in these Tables show that:

∗In the short-term trading: Δα (X8X) < Δα (Bitcoin) < Δα (HelloGold)< Δα (Gold)< Δα (Ethereum), indicating that the X8X cryptocurrency has the less multifractal structure among these markets in the short-term trading.

In addition, d (Bitcoin)< d (Gold) < d(Ethereum)<d(X8X)<d(HelloGold) implies that herding behavior is less intensive in HelloGold and high in the Bitcoin market in short-term trading. In other words, herding behavior is more intensive in conventional markets than in Sharia-compliant cryptocurrencies.

∗In the long-term trading: Δα (Ethereum) < Δα (X8X) < Δα (Bitcoin) < Δα (Gold) < Δα (HelloGold). Therefore, HelloGold cryptocurrency has the less multifractal structure among these cryptocurrencies in long-term trading.

Furthermore, d (Ethereum) < d(Bitcoin)<d(X8X)<d(HelloGold) < d (Gold) implies that the herding behavior is less intensive in Gold and high in the Ethereum markets in the long-term trading. In the same way, herding behavior is more present in conventional markets during the long horizon trade.

These results can be explained by the fact that conventional cryptocurrencies are mainly considered speculative assets and are subject to behavioral biases. In other words, these findings might be attributed to the fundamental structure of gold-backed cryptocurrencies as they are less exposed to speculation and behavioral biases.

In the second step of the proposed methodology, we tested the impact of COVID-19 on the studied cryptocurrencies. Table 5 depicts the efficiency and multifractality before and after the outbreak date. The ranking of these cryptocurrencies is determined based on the inefficiency index IEI. The findings in this table show that the Gold based markets were the most efficient markets before the outbreak. After the COVID-19 eruption, Ethereum is found to be the most efficient market.

**Table 5:** Efficiency and multifractality before and after the COVID 19 outbreak.

	Bitcoin		Ethereum		HelloGold		X8X		GOLD	
	Before	After	Before	After	Before	After	Before	After	Before	After
Δα	0.5189	**0.8909**	0.5316	**0.2902**	0.6138	**0.5871**	0.5462	**0.7438**	0.5319	**0.7658**
Δ H_q_	0.2917	**0.5597**	0.3196	**0.1386**	0.3578	**0.3251**	0.311	**0.4894**	0.3323	**0.483**
Hurst (mean)	0.6691	**0.6653**	0.7002	**0.6059**	0.4381	**0.4253**	0.4532	**0.4938**	0.5355	**0.5767**
D	1.3308	**1.3346**	1.2998	**1.394**	1.5618	**1.5746**	1.5467	**1.506**	1.4644	**1.4681**
IEI	0.1827	**0.2798**	0.1856	**0.095**	0.1789	**0.2935**	0.1555	**0.2447**	0.1661	**0.2415**
Rank	4	**4**	5	1	3	5	1	3	2	2

**Table 6:** Cryptocurrency prices and COVID-19 proxies.

		HelloGold	X8X	Bitcoin	Ethereum	Gold
Intercept	Estimate	0.00198	0.00096	0.00253	0.00399	-0.00016
	SD	0.00236	0.00254	0.0011	0.00139	0.00023
	p-value	0.400741	0.70591	0.01148	0.00421	0.49649
Return_-1_	Estimate	-0.262391	-0.18224	-0.12514	-0.1603	0.07243
	SD	0.080669	0.0593	0.07057	0.0696	0.05999
	p-value	0.00114	0.00211	0.07619	0.02132	0.22726
Growth cases	Estimate	31.80031∗∗	-14.03651∗∗	-1.51959∗∗	32.24001∗∗	-2.44599 ∗∗
	SD	13.18980	10.99517	7.55996	14.03644	2.04703
	p-value	0.01591	0.02017	0.08406	0.02162	0.02321
Growth deaths	Estimate	-0.04506∗∗	-0.01486∗	-0.02101∗	-0.05614	0.00457 ∗
	SD	0.04883	0.03941	0.03207	0.04317	0.00694
	p-value	0.03560	0.07059	0.05124	0.19349	0.05098
AR (1)		0.035	0.065	0.023	0.041	0.081
AR (2)		0.214	0.412	0.752	1.141	0.038
Hansen test		8.6	5.3	1.4	7.8	1.5

Signifiance codes: 0.01 ‘∗∗∗’ 0.05 ‘∗∗’ 0.1 ‘∗’.

Furthermore, all of these markets became less efficient after the pandemic outbreak except for the Ethereum market. Besides, we noted positive and negative effects of the pandemic on each cryptocurrency market.

To justify the impact of this crisis on these market responses and give more robustness to our results, we tested the effect of COVID-19 pandemic proxies on each cryptocurrency return. Table 6 illustrates the system GMM estimation results highlighting the influence of the pandemic proxies on the studied markets. In Table 6, the Arellano-Bond test for AR (1) and AR (2) tests show that there is no further serial correlation. Given the validity of the instrumental variables and the moment conditions provided by the System GMM (Hansen test), the estimates can be regarded as consistent and reliable.

The independent variables consisting of COVID-19 proxies are often explaining the Gold and the cryptocurrency returns. These results confirm our findings in Table 5. Besides, we notice that gold and gold-backed cryptocurrencies (X8X and HelloGold) present similar responses to the pandemic proxies.

This research finding is not surprising because X8X and HelloGold are backed by Gold, and their behavior would probably be related to the gold market.

These results corroborate previous studies that demonstrated the effect of the COVID-19 pandemic on financial markets (Yarovaya et al., 2020; Salim Lahmiri; Bekiros, 2020; Soofi et al., 2020).

Market efficiency theory announces that prices include all information relating to firms and converge at all times towards its fundamental value, thanks to rational investors' accurate sets of information, creating this equality between the asset's fundamental value and its selling price on the market. If relevant potential data only hits the offer and the demand, the cryptocurrency price represents its fundamental value (Fung et al., 2010).

However, in the presence of irrational investors, the errors identified would be revised by the arbitration mechanism, leading to the maintenance of the fundamental value. These investors focus on the relevance of public information circulating on the market to their private information held by other economic agents and the calculation of the incorporation speed and integration of data into the price of securities (Daniel and Titman, 1999). From a behavioral viewpoint, the decisions of individuals are possibly biased by emotions, sensations, heuristics, and mental states. Several academics consider that the market's instability, namely, the gap between the price and its fundamental value, results from low diversity of opinions. In a financial market, uncertainty on the fundamentals pushes the agents to seek relevant information concerning the assets based on the opinion of others, thus forming a collective thought. This situation of unanimity creates a disconnection of prices from their fundamental value to reflect only the average opinion of the market. Therefore, investors can contribute to excess volatility in operating markets, thereby adopting tracking strategies. Many empirical works reach the same inferences. Indeed, Tan et al. (2008) found that the herd behavior of investors amplifies the volatility of returns and transaction volumes by analyzing the Chinese stock market.

In summary, the lack of financial market efficiency can be justified by the presence of irrational investors and their response to the asymmetric information capable of creating herding behavior. Along these lines, herding behavior can cause excessive volatility, especially in crisis periods. Consequently, quantifying inefficiency and herding behavior helps detect extreme volatilities and market risks. The empirical findings of this study reach the same inference and align with the expectations based on behavioral theory.

In conclusion, the results of this study confirm the first hypothesis and disprove the second assumption. In other words, gold-backed cryptocurrencies were less inefficient before the pandemic and became more inefficient after and during the COVID-19 crisis. Along the same lines, the empirical findings of this research show that herding behavior became more considerable after the pandemic outbreak, except for the Ethereum market. These results are in accordance with previous studies on the COVID-19 pandemic effect on cryptocurrency markets (Salim Lahmiri and Bekiros, 2020), Gold (Kristoufek and Vosvrda, 2016), and gold-backed cryptocurrency markets (Yousaf and Yarovaya, 2022; Jalan et al., 2021) during the COVID-19 pandemic.

Furthermore, they are of great importance, especially for investors, marketers, and policymakers. In other words, investors avoiding risky assets will prefer X8X in the long-term horizon trade and Bitcoin in the short trade. Nevertheless, marketers choosing risky investments will opt for the other kinds of studied assets. In summary, gold-backed cryptocurrencies were less risky in long-term trades before the pandemic. In conclusion, as the pandemic hits all the types of the studied markets, traders and policymakers might search for safe-haven assets capable of reducing the pandemic risks.

## 6 Conclusion

This work aimed to study efficiency and detect herding behavior in cryptocurrency and gold markets. The gold-backed, conventional cryptocurrencies and Gold were used to illustrate the method and focus on the influence of the COVID-19 pandemic on the performance of gold, conventional, and gold-backed cryptocurrencies. Therefore, we compared their efficiency before and during the pandemic and tested the effect of the pandemic proxies on these markets’ prices. According to the empirical results of the GHE calculation, most cryptocurrencies are multifractal in the long horizon and short trading scale. Consequently, their prices and their returns are predictable. For more robust results, we measured the efficiency level of these cryptocurrencies by employing an inefficiency index (IEI). The results prove that the gold-backed cryptocurrency market (X8X) is the most efficient for long-term trading. However, the conventional cryptocurrency market (Bitcoin) is the most efficient in the short horizon. The presented degree of efficiency may help cryptocurrency traders establish their trading strategies. These markets may be the best option for marketers who would rather avoid risks in their respective trade horizons. The conventional cryptocurrency (Ethereum) may be the best alternative for those who prefer risk-taking, as it is the most inefficient in short-scale trading.

Thus, the efficiency study is a tool and a guide for market policymakers to make the best choice. Furthermore, gold-backed cryptocurrencies (X8X and the HelloGold) are the most efficient markets before the outbreak. Besides, gold-backed cryptocurrency markets present a smaller level of herding behavior than conventional cryptocurrencies on tall scales.

In addition, efficiency decreased after the outbreak for each market except for Ethereum. The empirical results show that most COVID-19 proxies have an evident impact on each cryptocurrency's dynamics. Besides, the research findings indicate that gold markets present a similar response to the gold-backed cryptocurrency markets. In other words, gold-backed cryptocurrency and gold prices fluctuated similarly after the pandemic outbreak leading to close investor behavior in these markets during the COVID-19 pandemic. These findings have pertinent implications for Sharia-compliant investment. First, they showed that gold-backed cryptocurrency series are predictable even during pandemics, high volatility, and instability. Second, market inefficiency highlights the presence of behavioral biases and market imperfections. This work, therefore, justifies these issues by quantifying the herding bias during different time scales and crisis periods. Third, the achieved results of this study showed that the available information is primarily reflected in the gold-backed cryptocurrency (X8X) prices as it represents the most efficient market in the long trading horizon and the conventional cryptocurrency market (Bitcoin) on the short scale. Sharia boards and financial authorities must improve the transparency degree and risks of gold-based cryptocurrencies to make them more compatible with Sharia laws.

Unlike political and social events like wars, the recent coronavirus COVID-19 is a biological disaster that damages human health and the economic sphere leading to spillover and market reaction. Gold-backed cryptocurrency markets have different characteristics and behaviors, leading to varying responses than conventional cryptos and similar responses to Gold during pandemics. From a future perspective, further analysis of these findings might be necessary to exploit stablecoins policies to mitigate market risks and build hedging strategies.

## Declarations

### Author contribution statement

Emna Mnif, Bassem Salhi, Lotfi Trabelsi and Anis Jarboui: Conceived and designed the experiments; Performed the experiments; Analyzed and interpreted the data; Contributed reagents, materials, analysis tools or data; Wrote the paper.

### Funding statement

This research did not receive any specific grant from funding agencies in the public, commercial, or not-for-profit sectors.

### Data availability statement

Data will be made available on request.

### Declaration of interest's statement

The authors declare no conflict of interest.

### Additional information

No additional information is available for this paper.
